# Combined Effect of Salicylic Acid and Proline Mitigates Drought Stress in Rice (*Oryza sativa* L.) through the Modulation of Physiological Attributes and Antioxidant Enzymes

**DOI:** 10.3390/antiox12071438

**Published:** 2023-07-18

**Authors:** Tahmina Akter Urmi, Md. Moshiul Islam, Kamrun Naher Zumur, Md. Anwarul Abedin, M. Moynul Haque, Manzer H. Siddiqui, Yoshiyuki Murata, Md. Anamul Hoque

**Affiliations:** 1 Department of Soil Science, Faculty of Agriculture, Bangladesh Agricultural University, Mymensingh 2202, Bangladesh; urmi.bsmrau@gmail.com (T.A.U.); m.a.abedin@bau.edu.bd (M.A.A.); 2 Department of Agronomy, Faculty of Agriculture, Bangabandhu Sheikh Mujibur Rahman Agricultural University, Gazipur 1706, Bangladesh; kamrunnaherzumur123@gmail.com (K.N.Z.); moynul60@bsmrau.edu.bd (M.M.H.); 3 Graduate School of Environmental, Life, Natural Science and Technology, Okayama University, 1-1-1 Tsushima-Naka, Okayama 700-8530, Japan; muta@cc.okayama-u.ac.jp; 4 Department of Botany and Microbiology, College of Science, King Saud University, Riyadh 11451, Saudi Arabia; mhsiddiqui@ksu.edu.sa

**Keywords:** rice, drought stress, osmolytes, reactive oxygen species, lipid peroxidation, antioxidant

## Abstract

Salicylic acid (SA) and proline exhibit protective effects against a wide range of stresses. However, the combined impact of SA and proline on rice under drought stress is still unknown. Therefore, we investigated the protective roles of SA and/or proline in conferring drought tolerance in rice. There were eight treatments comprising the control (T1; 95–100% FC), 1.5 mM SA (T2), 2 mM proline (T3), 0.75 mM SA + 1 mM proline (T4), 45–50% FC (T5, drought stress), T5 + 1.5 mM SA (T6), T5 + 2 mM proline (T7), and T5 + 0.75 mM SA + 1 mM proline (T8), and two rice varieties: BRRI dhan66 and BRRI dhan75. Drought stress significantly decreased the plant growth, biomass, yield attributes, photosynthetic rate (Pn), stomatal conductance (gs), transpiration rate (Tr), photosynthetic pigments (chlorophyll and carotenoids content), relative water content (RWC), membrane stability index (MSI), soluble sugar and starch content, and uptake of N, P and K^+^ in roots and shoots. Drought-induced oxidative stress in the form of increased hydrogen peroxide (H_2_O_2_) production and lipid peroxidation (MDA) was observed. The combined application of SA (0.75 mM) + proline (1 mM) was found to be more effective than the single application of either for drought stress mitigation in rice. A combined dose of SA + proline alleviated oxidative stress through boosting antioxidant enzymatic activity in contrast to their separate application. The application of SA + proline also enhanced proline, soluble sugar and starch content, which resulted in the amelioration of osmotic stress. Consequently, the combined application of SA and proline significantly increased the gas exchange characteristics, photosynthetic pigments, RWC, MSI, nutrient uptake, plant growth, biomass and yield of rice. Therefore, the combined application of SA and proline alleviated the detrimental impacts of drought stress more pronouncedly than their separate application did by increasing osmoprotectants, improving nutrient transport, up-regulating antioxidant enzyme activity and inhibiting oxidative stress.

## 1. Introduction

Rice (*Oryza sativa* L.) is the staple food for nearly half of the world’s population, especially people in developing countries. Approximately 160 million hectares of land in the globe is used for rice cultivation, mostly in Asia, where about 46.6% of the world’s population resides [1]. The world’s population is increasing more sharply than ever before. Global agriculture feeds more than seven billion people and this number is expected to increase to nine billion by the year 2050 [2]. Therefore, it is an crucial requirement to increase the rice production by several times to fulfil the food requirement for this over-growing population. However, the availability of agricultural land is decreasing regularly due to its rapid and non-judicious use for modernization and biotic and abiotic stresses [3]. Moreover, the current status of climate change has led to higher temperatures, prolonged dry spells and severe droughts which negatively impact rice production [4,5]. Globally water shortage is becoming a critical issue. Approximately 69% of freshwater in the globe is used for agriculture [6]. Rice cultivation is dominated in the world where the loss of a huge amount of ground water is of great concern for future agriculture. Drought has exaggerated for about 40% of the world’s population while near 700 million people are at risk of being displaced as a result of drought by 2023 [7].

Compared to other crops, rice is more susceptible to drought. Drought causes numerous changes at the physiological, metabolic and molecular levels, hence seriously influencing the growth and development of rice [8]. However, plant response to drought stress is different in terms of their morphology and physiology under drought conditions. Leaf area, cell size and the intercellular volume of crops reduce due to water stress [9]. Lower soil moisture due to drought stress led to a lower water content in leaves causing guard cells to lose turgor pressure, hence causing a reduction in the size of stomatal pores [10] and/or causing stomatal closure. Drought reduces plant productivity by inhibiting growth and photosynthesis [10]. A positive correlation between photosynthesis and crop yield is commonly found. For survival or regrowth under drought stress, plants activate different mechanisms such as the minimization of water loss through reducing leaf area and stomatal closure or the maximization of water uptake capacity via deep, dense root systems, changes in cell wall elasticity and the maintenance of osmotic adjustment (OA) [11]. The accumulation of organic substances and inorganic ions by plants exposed to water stress is believed to be a mechanism participating in OA which could promote drought stress tolerance in plants [12]. 

Drought causes the overproduction of reactive oxygen species (ROS). Reactive oxygen species alter the metabolic and oxidative homeostasis of plant cells, hence promoting membrane lipid peroxidation [13,14]. The accumulation of excessive ROS can lead to leaf senescence through the breakdown of photosynthetic pigments [13,14,15]. Plants respond to stress stimuli immediately after stress exposure and make physiological and biochemical changes through signal transduction for survival and growth [16]. Thus, plants produce secondary metabolites and different compatible osmoprotectants such as proline, soluble sugar, and starch, which helps to regulate plant physiological processes, and stabilize enzyme and membrane integrity under drought stress [17,18]. Plants generally remobilize starch to provide energy and carbon when photosynthesis may be potentially limited due to lower water supply conditions under drought [19]. Furthermore, plants possess antioxidants, both enzymatic and non-enzymatic, against increased oxidative stress to maintain a cellular redox equilibrium by scavenging ROS [14,16]. 

Various approaches have been developed to overcome the drought problem. Obviously, the improvement of drought-tolerant genotypes has been proposed as the most effective way to reduce the deleterious effects of drought on crop production. However, genetic self-defense ability is not adequate to fully rescue plants from stress damage. That is why plant researchers are searching for alternatives to enhance plants’ ability to survive under adverse environmental conditions. Recently, plant growth regulators and osmoprotectants have been found to play a central role in the integration of the responses expressed by plants under stress conditions. Salicylic acid (SA) is a ubiquitous phenolic phytohormone and an essential part of plant defense mechanisms. It plays a vital role in many of the physiological processes of plants [20,21]. SA reduced the drought-induced inhibition of seed germination in rice [16]. The supplementation of SA to drought-stressed crops notably increased stomatal conductance and eventually increased the net CO_2_ assimilation rate and plant growth [22]. Exogenous SA application increased plant height and dry mass while decreasing the membrane lipid peroxidation of drought-stressed wheat. Ionic leakage and the accumulation of toxic ions were significantly reduced due to SA application [23]. It has been reported that the exogenous application of SA decreases oxidative stress in drought-stressed plants by modulating important enzymatic and non-enzymatic pathways and also the glyoxalase system [22]. In many plant species, the increased accumulation of proline was observed as an indicator of stress tolerance [24,25,26,27]. However, most plants, especially under elevated levels of stress, cannot synthesize a sufficient amount of these osmoregulators. Recently, the exogenous application of proline revealed that it could act as a protectant under drought stress [28,29,30]. Apart from osmoprotection, proline also can eliminate oxidative stress by triggering the antioxidant defense and also glyoxalase system [28,30,31]. Proline stabilizes sub-cellular structure and cellular redox buffering capacity [32]. However, many aspects of exogenous SA- and/or proline-mediated drought tolerance in rice remain elusive. With this background, the present research work was, therefore, performed to analyze the potential roles and possible mechanisms of SA- and/or proline-mediated drought stress tolerance in rice. The means of amelioration of drought with a better understanding of drought-tolerant plant characteristics will be useful for the varietal improvement of the crop which can be useful in drought stress conditions under the context of the climate change situation of the globe.

## 2. Materials and Methods

### 2.1. Plant Materials and Treatments

Two rice (*Oryza sativa* L.) varieties—BRRI dhan66 (high-yielding; drought-tolerant) and BRRI dhan75 (high-yielding; drought-susceptible)—were collected from Plant Breeding Division, Bangladesh Rice Research Institute (BRRI), Gazipur, Bangladesh [33]. The experiment was conducted at the vinyl house. The temperature, relative humidity and light period of the vinyl house was 25 ± 2 °C, 65–70%, and 16 h, respectively, with a 80 µmol m^−2^ s^−1^ photon flux density and 8 h dark period. The seeds were surface-sterilized with 0.2% HgCl_2_ solution for 5 min, washed thoroughly with distilled water several times and sown in the plastic tray filled with soil for germination. Twenty-one-day-old rice seedlings were transferred from the plastic tray to 20 L plastic pots. Two rice varieties, two soil moisture levels, a 95–100% field capacity (FC), and 45–50% FC with or without 1.5 mM salicylic acid (SA) (Wako, Japan) and/or 2 mM proline (L (-) Proline, Wako, Japan) were used as treatment variables. The levels of SA and proline were selected based on a preliminary laboratory study (Table S1). Therefore, the treatment combinations were as follows: control (T1; 95–100% FC); 1.5 mM SA (T2); 2 mM Proline (T3); 0.75 mM SA + 1 mM proline (T4); 45–50% FC (T5); T5 + 1.5 mM SA (T6); T5 + 2 mM proline (T7); T5 + 0.75 mM SA + 1 mM proline (T8). The drought treatments were started 28 days after transplantation (DAT). Salicylic acid and/or proline was sprayed using a hand atomizer 15, 30, 45 and 60 days after transplantation on drought-treated plants. Control plants were sprayed with distilled water (DW). Every time, a 100 mL solution of SA and/or proline or distilled water was applied to each plant. Tween-20 at a concentration of 0.05% (*v*/*v*) was used as a wetting agent with SA, proline and distilled water. The experiment was conducted in a factorial completely randomized design with five replications.

### 2.2. Pot Preparation and Fertilizer Application

Soil form the Codda area of Gazipur district was used in this pot experiment. The soil was silty clay loam in texture with a bulk density (g/cc) of 1.36, particle density (g/cc) of 2.61, soil pH of 5.94, organic carbon concentration (%) of 0.98, total nitrogen concentration (%) of 0.093, an available P amount (mg kg^−1^ soil) of 18.86, exchangeable K (meq/100 g soil) amount of 0.128, available S amount of (mg kg^−1^ soil) 20.91, and field capacity of 30.55% vol/vol. The size of the pot was 24 cm (diameter) × 30 cm (height). Each pot was filled with 13 kg of soil mixed with cow dung (1:0.25 ratio). The average monthly maximum and minimum air temperature of this area varies between 28 °C to 36 °C and 19 °C to 26 °C. Uniform distance was maintained between each pot. Fertilizers were incorporated in the soil in amounts of 1.28, 0.46, 0.68, 0.22, 0.079 g of urea, triple super phosphate (TSP), muriate of potash (MoP), gypsum and zinc sulphate per pot, respectively [33]. The total amount of TSP, MP, gypsum, zinc sulphate and one-third of urea was applied at the time of final pot preparation. The remaining urea was applied in two splits. The first split was applied at the maximum tillering stage and the other was applied at the panicle initiation stages of rice.

### 2.3. Treatment Imposition

After seedling establishment, one healthy plant was kept in each pot for subsequent treatment imposition. During treatment imposition, 95–100% and 45–50% FC were maintained for control and drought stress, respectively. Water was applied to bring the soil moisture to the higher range of each treatment (50 and 100% FC). Subsequent irrigation was applied when the soil moisture came down to the lower levels (45 and 95% FC) of those treatments, respectively. Soil moisture status (%) (*v*/*v*) under different treatments involving ranges of FC was monitored in 5 day intervals from 28 DAT until maturity. Irrigation water was applied using a measuring cylinder. A soil moisture meter (Stevens, Field POGO, Portland, OR, USA) was used to assess the field capacity of the soil. 

### 2.4. Assessment of Growth and Yield Parameters

Plant samples were collected after six (6) weeks of treatment imposition from five replications for dry matter partitioning. The data on plant height, fresh weight and dry weight per plant were recorded individually from all treatments. The collected plant samples were air-dried under room-temperature conditions first and then oven-dried at 70 °C for 72 h and then the dry weight per plant was recorded. At the time of harvest (110 DAT), the data on yield components, such as plant height, total tillers per hill, effective tillers per hill, panicle length, filled grain per panicle, unfilled grain per panicle, thousand seed weight and seed yield per hill, were recorded.

### 2.5. Gas Exchange Characteristics 

Gas exchange measurements such as those of stomata conductance (gs), transpiration rate (Tr), and photosynthetic rate (Pn) were recorded after four weeks of treatment imposition. The uppermost leaves of each variety of all the treatments were used in gas exchange measurements. The Li-COR, 6400 portable photosynthetic system (Li-COR, Lincon, NE, USA) was used for gas exchange measurements.

### 2.6. Estimation of Total Chlorophyll and Carotenoid Content

Total chlorophyll and carotenoid content from fresh leaves were determined on a fresh weight basis and extracted with 80% acetone using a double-beam spectrophotometer according to Fadeel [34] after four weeks of treatment imposition. 

### 2.7. Estimation of Leaf Relative Water Content and Membrane Stability Index 

After four weeks of treatment imposition, the relative water content (RWC) of leaves was measured as described previously by Fairoj et al. [35]. The following formula was used for RWC calculation:

RWC = [Fresh weight − Dry weight] ÷ [Turgid weight − Dry weight] × 100

The membrane stability index (MSI) was determined after four weeks of treatment imposition using 200 mg of fresh leaf tissue as described previously by Rady [36]. Two sets of samples were prepared. The first set was incubated for 30 min at 40 °C in a water bath, and the electrical conductivity (EC_1_) of the solution was recorded. The second set was incubated for 10 min at 100 °C in a water bath, and electrical conductivity (EC_2_) was measured. MSI was calculated using the following formula:

MSI (%) = [1 − (EC_1_/EC_2_)] × 100

### 2.8. Osmoprotectant Measurements

Proline content was measured as described previously by Sen et al. [37]. A determination of the soluble sugar and starch content in the leaves of the rice was conducted in accordance with the method of Du et al. [38].

### 2.9. Hydrogen Peroxide and Melondealdehyde Determination

Hydrogen peroxide (H_2_O_2_) and malondialdehyde (MDA) content were determined as described previously by Islam et al. [3].

### 2.10. Estimation of Antioxidant Enzymatic Activity 

The fresh leaf tissue was homogenized in a deep-freezer-cooled pestle and mortar in the presence of 1 mL of ice-cold 100 mM potassium phosphate buffer (pH 7.0) containing 1% of polyvinyl pyrrolidone. After centrifuging the homogenates at 12,000× *g* for 30 min at 4 °C, the supernatant was collected and used to determine different enzyme activities. Catalase (CAT, EC: 1.11.1.6) activity was determined according to the method of Islam et al. [3] through monitoring the decrease in absorbance at 240 nm for 1 min caused by the decomposition of H_2_O_2_ with a spectrophotometer (Shimadzu, UV-1201; 1, Nishinokyo Kuwabara-cho, Nakagyo-ku, Kyoto 604-8511, Japan). Ascorbate peroxidase (APX, EC: 1.11.1.11) activity was determined in accordance with the procedure used by Islam et al. [3]. The reaction mixture for the peroxidase contained 50 mM potassium phosphate, at pH 7.0, 0.5 mM ascorbate, 0.1 mM hydrogen peroxide and 0.1 mM ethylenediaminetetraacetic acid (EDTA) in a total volume of 1 mL. The H_2_O_2_-mediated oxidation of ascorbate was calculated from the decrease in absorbance at 290 nm min^−1^ when the extinction coefficient was 2.8 mM^−1^ cm^−1^ with a spectrophotometer (Shimadzu, UV-1201; 1, Nishinokyo Kuwabara-cho, Nakagyo-ku, Kyoto 604-8511, Japan). Guaiacol peroxidase (GPX, EC 1.11.1.7) activity was assessed via the method of Nakano and Asada [39] using guaiacol as a substrate. The reaction mixture contained 0.6 mL of 50 mM potassium phosphate (pH 8.0), 0.1 mL of EDTA, 0.1 mL of H_2_O_2_, and 0.1 mL guaiacol. The reaction was initiated by adding 0.1 mL of the enzyme extract. The absorbance was recorded at 470 nm when the extinction coefficient was 26.6 mM^−1^ cm^−1^ with a spectrophotometer (Shimadzu, UV-1201; 1, Nishinokyo Kuwabara-cho, Nakagyo-ku, Kyoto 604-8511, Japan). Superoxide dismutase (SOD, EC: 1.15.1.1) activity was assayed using the method of Dhindsa and Matowe [40]. The reaction mixture contained 1.5 mL of 100 mM phosphate buffer (pH 7.4), 0.1 mL of 50 μM riboflavin, 0.2 mL of 10 mM methionine, 100 μLof 1 mM ethylenediaminetetraacetic acid (EDTA) with equal amounts of enzyme extract, and 70 μM nitro blue tetrazolium chloride (NBT). The reaction mixture was kept in fluorescent tubes for 20 min. The absorbance was measured at 560 nm with a spectrophotometer (Shimadzu, UV-1201; 1, Nishinokyo Kuwabara-cho, Nakagyo-ku, Kyoto 604-8511, Japan).

### 2.11. Measurements of Nutrient Content

The roots and shoots of harvested rice were collected from each pot. The collected roots and shoots samples were air-dried in the room condition first and then oven-dried at 70 °C for 72 h after which they were ground with a grinding machine. A 20-mesh sieve was used to sieve the ground samples. The root and shoot samples were analyzed for N, P, and K. Kjeldhal systems [41] were used for the determination of the total nitrogen of the plant samples, while the acid digestion method [42,43] was used for the determination of total P and K.

### 2.12. Statistical Analysis

The observed data were evaluated statistically using ‘Statistix version 10’ software. The data were analyzed with the analysis of variance (ANOVA) technique. Comparison of the mean difference was performed using Tukey’s test (*p* < 0.05).

## 3. Results

### 3.1. Growth and Biomass of Rice 

Drought stress significantly suppressed the plant height of both the rice varieties (Table 1). Drought stress decreased the plant height of drought-tolerant BRRI and drought-susceptible BRRI dhan75 by 8.2% and 29.5%, respectively, compared to that of the control plants. The exogenous application of salicylic acid (SA) and/or proline to drought-stressed rice significantly enhanced the plant height of both the rice varieties. The exogenous application of SA to drought-stressed BRRI dhan66 and BRRI dhan75 enhanced plant height by 2.3% and 15.7%, respectively, while the exogenous application of proline enhanced plant height by 1.3% and 15.2%, respectively, compared to that with drought stress alone. However, a combined dose of SA + proline applied to drought-stressed BRRI dhan66 and BRRI dhan75 enhanced shoot length by 5.1% and 25.8%, respectively. 

Drought stress significantly decreased the fresh weight (FW) per plant of both rice varieties (Table 1). Drought-stressed rice treated with SA and/or proline exhibited higher FW compared to rice plants treated with drought alone.

However, the maximum increases in FW of BRRI dhan66 (15.0%) and BRRI dhan75 (103.3%) were observed for the combined application of SA + proline under drought stress conditions. It is apparent from Table 1 that drought stress significantly reduced the dry weight (DW) per plant of both the rice varieties by 12.5% for drought-tolerant BRRI dhan66 and 31.8% for drought-susceptible BRRI dhan75 compared to that of control plants. The exogenous application of SA and proline to drought-stressed rice enhanced the DW of drought-tolerant BRRI dhan66 by 4.5% and 3.8%, respectively, and drought-susceptible BRRI dhan75 by 17.5% and 15.5%, respectively. However, the combined application of SA + proline to drought-stressed rice enhanced the DW of drought-tolerant BRRI dhan66 by 7.5% and that of drought-susceptible BRRI dhan75 by 24.3%. 

### 3.2. Agro-Morphology and Yield of Rice

Th plant height of rice showed significant differences in response to drought, SA and/or proline (Table 2). Table 2 shows that drought stress decreased the plant height of BRRI dhan66 and BRRI dhan75 by 8.0% and 41.2%, respectively, compared to that of the control plants. However, the exogenous application of SA and/or proline to drought-stressed rice significantly increased plant height. The combined treatment of SA + proline to drought-stressed plants increased the plant height of BRRI dhan66 and BRRI dhan75 by 5.2% and 28.2%, respectively, compared to that of plants treated with drought alone. 

Total tillers per hill and effective tillers per hill of BRRI dhan66 and BRRI dhan75 were significantly decreased due to drought stress (Table 2). Drought-stressed rice pre-treatments with SA and/or proline exhibited a higher number of total tillers per hill and effective tillers per hill compared to those of only stressed rice. However, the maximum increases in total tillers per hill and effective tillers per hill were observed for the combined application of SA + proline to drought-stressed rice, which were 12.1% and 42.7% of the total tillers per hill for BRRI dhan66 and BRRI dhan75, respectively, and 11.1% and 53.0% of the effective tillers per hill for BRRI dhan66 and BRRI dhan75, respectively, compared to those of drought-stressed rice alone. 

Table 2 shows that drought stress decreased the panicle length of BRRI dhan66 and BRRI dhan75 by 15.0% and 49.2%, respectively, compared to that of the control plants. On the other hand, the exogenous application of SA and/or proline to drought-stressed rice did not significantly increase the panicle length. However, the combined dose of SA + proline to drought-stressed plants increased the panicle length of BRRI dhan66 and BRRI dhan75 by 4.6% and 41.0%, respectively, compared to that of plants treated with drought alone. Drought stress decreased the number of filled grains per panicle of BRRI dhan66 and BRRI dhan75 by 17.6% and 68.0%, respectively, and increased the number of unfilled grains per panicle by 141.2% and 314.2%, respectively, compared to those of control plants (Table 2). Th exogenous application of SA and/or proline to drought-stressed rice significantly increased the number of filled grains per panicle and decreased the number of unfilled grains per panicle of both the varieties. However, statistically, the maximum numbers of filled grains per panicle and the minimum number of unfilled grains per panicle were observed during the combined application of SA + proline to drought-stressed rice (Table 2). 

Drought stress reduced the thousand seed weight of drought-tolerant BRRI dhan66 and drought-susceptible BRRI dhan75 by 10.2% and 36.7%, respectively, compared to that of the control plants (Table 2). The exogenous application of SA and/or proline to drought-stressed rice significantly increased the thousand seed weight of both the rice varieties. However, the maximum increases in thousand seed weight were observed for the combined application of SA + proline to drought-stressed rice, these being 7.2% and 28.3% for BRRI dhan66 and BRRI dhan75, respectively, compared to those of drought-stressed rice alone.

Drought stress significantly decreased the seed yield per hill of both drought-tolerant and drought-susceptible rice varieties (Table 2). Drought stress reduced the seed yield per hill of BRRI dhan66 and BRRI dhan75 by 26.9% and 80.2%, respectively, compared to that of the control. The exogenous application of SA and/or proline to drought-stressed rice enhanced the seed yield per hill. However, the maximum increases in seed yield per hill were found for the combined application of SA + proline to drought-stressed rice, these being 19.1% and 130.6% for BRRI dhan66 and BRRI dhan75, respectively, compared to those of drought-stressed rice alone.

### 3.3. Gas Exchange Characteristics and Photosynthetic Pigments of Rice

Drought stress significantly reduced the photosynthetic rate (Pn) of both the rice varieties, which was 29.0% and 59.3% lower for BRRI dhan66 and BRRI dhan75, respectively, compared to that of the control (Table 3). The foliar application of SA and/or proline to drought-stressed rice significantly increased the Pn rate via the amelioration of the harmful effect of drought. However, the maximum Pn rates of drought-stressed BRRI dhan66 (13.8 µmol m^−2^ s^−1^) and BRRI dhan75 (10.5 µmol m^−2^ s^−1^) were observed in the T8 (drought stress + 0.75 mM SA + 1 mM proline) treatment, these being 23.7% and 71.2% higher for BRRI dhan66 and BRRI dhan75, respectively, compared to that of rice plants treated with drought alone.

Drought stress significantly reduced the stomatal conductance (gs) of both drought-tolerant BRRI dhan66 and drought-susceptible BRRI dhan75; hence, the lowest stomatal conductance values of BRRI dhan66 (0.276 mmol m^−2^ s^−1^) and BRRI dhan75 (0.106 mmol m^−2^ s^−1^) were obtained under drought stress (T5), which were 33.3% lower for BRRI dhan66 and 74.2% lower for BRRI dhan75 compared to those of the control plants (Table 3). Due to the amelioration of drought stress through the exogenous application of SA and/or proline, the highest stomatal conductance values of drought-stressed BRRI dhan66 (0.351 mmol m^−2^ s^−1^) and BRRI dhan75 (0.229 mmol m^−2^ s^−1^) were recorded for the T8 (drought stress + 0.75 mM SA + 1 mM proline) treatment, which were 27.2% and 116.0% higher for BRRI dhan66 and BRRI dhan75, respectively, compared to those of plants treated with drought alone. 

The transpiration rate (Tr) of rice varieties varied significantly due to drought and the exogenous application of SA and/or proline (Table 3). The lowest transpiration rates of BRRI dhan66 (4.06 mmol m^−2^ s^−1^) and BRRI dhan75 (1.89 mmol m^−2^ s^−1^) were obtained under drought stress (T5), which were 38.3% and 70.7% lower for BRRI dhan66 and BRRI dhan75, respectively, compared to those of the control plants. On the other hand, the foliar application of SA and/or proline to drought-stressed rice significantly increased the transpiration rate by ameliorating the harmful effect of drought. However, the maximum transpiration rates of drought-stressed BRRI dhan66 (5.04 mmol m^−2^ s^−1^) and BRRI dhan75 (3.65 mmol m^−2^ s^−1^) were observed for the T8 (drought stress + 0.75 mM SA + 1 mM proline) treatment, which were 24.1% and 93.1% higher for BRRI dhan66 and BRRI dhan75, respectively, compared to those of plants treated with drought alone.

**Figure 1:**
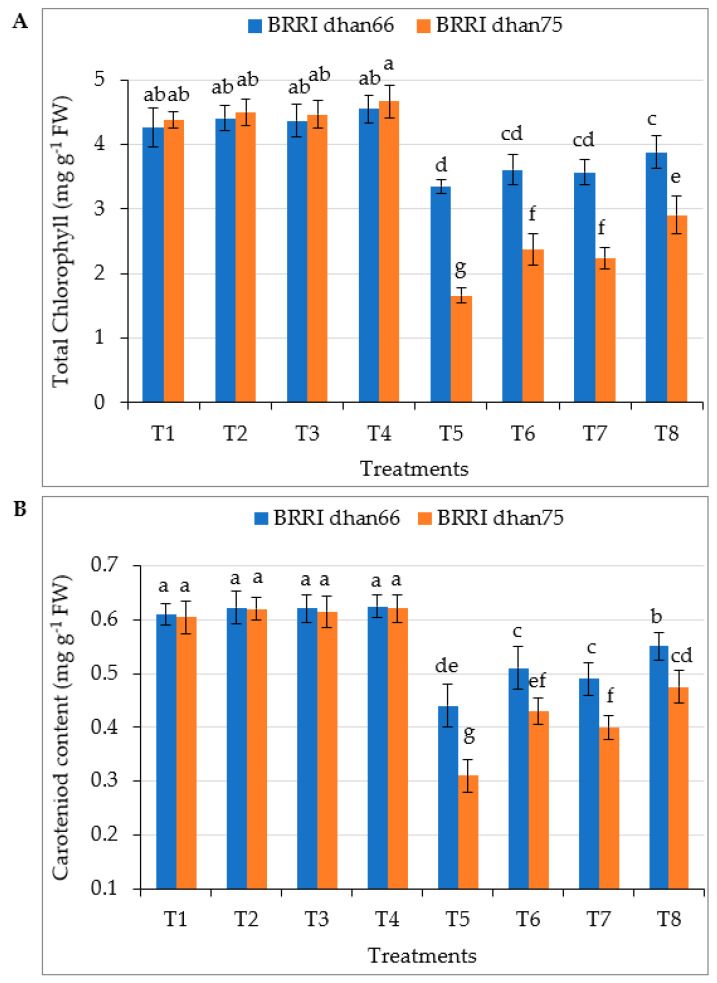
Foliar application of salicylic acid and/or proline regulating (**A**) total chlorophyll content and (**B**) carotenoid content of rice under drought stress. The vertical bar indicates the means of five replicates (*n* = 5) and the error bar indicates the standard errors. Different letters indicate significant differences at *p* ≤ 0.05 (Tukey’s test). T1 = control, T2 = 1.5 mM SA, T3 = 2 mM proline, T4 = 0.75 mM SA + 1 mM proline, T5 = 45–50% FC (drought stress), T6 = T5 + 1.5 mM SA, T7 = T5 + 2 mM proline, and T8 = T5 + 0.75 mM SA + 1 mM proline.

Drought stress significantly reduced the formation of chlorophyll in both the rice varieties, the values of which were 21.4% and 62.1% lower for BRRI dhan66 and BRRI dhan75, respectively, compared to that of the control (Figure 1A). The exogenous application of SA and/or proline enhanced the total chlorophyll content through the mitigation of drought stress. However, the maximum total chlorophyll contents of drought-stressed BRRI dhan66 (3.88 mg g^−1^ FW) and BRRI dhan75 (2.91 mg g^−1^ FW) were recorded for the T8 (drought stress + 0.75 mM SA + 1 mM proline) treatment, these being 15.8% and 75.3% higher for BRRI dhan66 and BRRI dhan75, respectively, compared to that of rice plants treated with drought alone. 

Carotenoid content decreased by 27.87% and 48.76% in drought-treated BRRI dhan66 and BRRI dhan75, respectively, compared to that of the control (Figure 1B). The exogenous application of SA and/or proline to drought-stressed rice significantly increased carotenoid content in both the varieties. However, the highest carotenoids contents of drought-stressed BRRI dhan66 (0.551 mg g^−1^ FW) and BRRI dhan75 (0.475 mg g^−1^ FW) were recorded for the T8 (drought stress + 0.75 mM SA + 1 mM proline) treatment, these being 25.2% and 53.2% higher for BRRI dhan66 and BRRI dhan75, respectively, compared that of rice plants treated with drought alone.

### 3.4. Relative Water Content and Membrane Stability Index

Drought stress significantly decreased the relative water content (RWC) of both the rice varieties (Table 3). Drought stress reduced the RWC of BRRI dhan66 and BRRI dhan75 by 15.7% and 35.6%, respectively, compared to that of the control. Salicylic acid and/or proline exhibited better performance in terms of RWC through mitigating the deleterious effect of drought stress. However, the highest RWC values of drought-stressed BRRI dhan66 at T5 (84.3%) and BRRI dhan75 (76.6%) were recorded for the T8 (drought stress + 0.75 mM SA + 1 mM proline) treatment, these being 10.3% and 33.6% higher for BRRI dhan66 and BRRI dhan75, respectively, compared to that of rice plants treated with drought alone.

**Figure 2:**
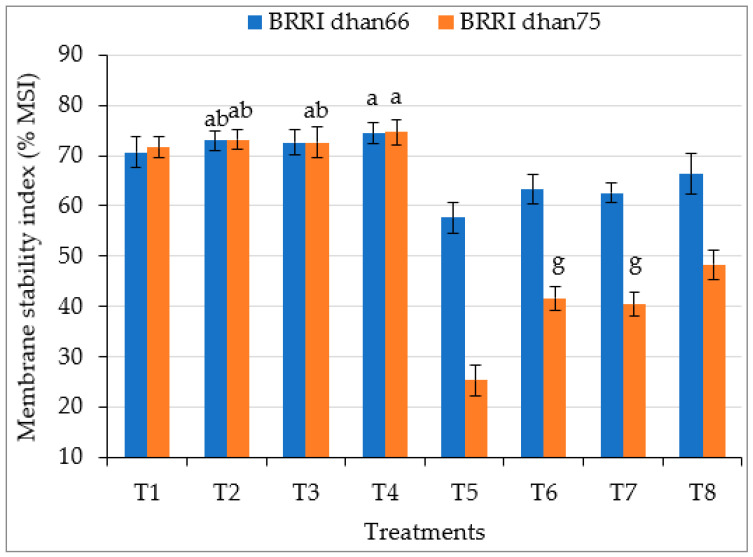
Foliar application of salicylic acid and/or proline regulating the membrane stability index (%MSI) of rice under drought stress. The vertical bar indicates the means of five replicates (*n* = 5) and the error bar indicates the standard errors. Different letters indicate significant differences at *p* ≤ 0.05 (Tukey’s test). T1 = control, T2 = 1.5 mM SA, T3 = 2 mM proline, T4 = 0.75 mM SA + 1 mM proline, T5 = 45–50% FC (drought stress), T6 = T5 + 1.5 mM SA, T7 = T5 + 2 mM proline, and T8 = T5 + 0.75 mM SA + 1 mM proline.

Drought stress significantly decreased the membrane stability index (% MSI) of both the rice varieties, this being 18.4% and 50.6% lower for drought-tolerant BRRI dhan66 and drought-susceptible BRRI dhan75, respectively, compared to that of control plants (Figure 2). This deleterious effect of drought was ameliorated via the exogenous application of SA and/or proline. However, the highest MSI values of drought-stressed BRRI dhan66 (66.38%) and BRRI dhan75 (48.36%) were recorded for the T8 (drought stress + 0.75 mM SA + 1 mM proline) treatment. 

### 3.5. Proline, Soluble Sugar and Starch Content

Drought stress significantly increased proline content in both the rice varieties (Table 4). The exogenous application of SA and/or proline to drought-stressed plants further significantly increased proline content in both the varieties. However, statistically, the highest proline content of drought-stressed BRRI dhan66 (4.25 µg g^−1^ FW) and BRRI dhan75 (3.78 µg g^−1^ FW) was observed for the T8 (drought stress + 0.75 mM SA + 1 mM proline) treatment, this being 13.3% and 31.3% higher for BRRI dhan66 and BRRI dhan75, respectively, compared to that of rice plants treated with drought alone.

Drought stress decreased the soluble sugar content of drought-tolerant BRRI dhan66 and drought-susceptible BRRI dhan75 by 19.0% and 32.8%, respectively, compared to that of control plants (Table 4). On the other hand, the foliar application of SA and/or proline to drought-stressed rice significantly increased the soluble sugar content of both the rice varieties. However, statistically, the highest soluble sugar content of drought-stressed BRRI dhan66 (29.6 mg g^−1^ FW) and BRRI dhan75 (25.3 mg g^−1^ FW) was observed for the T8 (drought stress + 0.75 mM SA + 1 mM proline) treatment, which was 15.6% and 24.6% higher for BRRI dhan66 and BRRI dhan75, respectively, compared to that of rice plants treated with drought alone. 

Drought stress reduced the starch content of drought-tolerant BRRI dhan66 and drought-susceptible BRRI dhan75 by 9.7% and 35.5%, respectively, compared to that of control plants (Table 4). During the amelioration of drought stress through the exogenous application of SA and/or proline, the highest starch content of drought-stressed BRRI dhan66 (15.5 mg g^−1^ FW) and BRRI dhan75 (13.9 mg g^−1^ FW) was recorded for the T8 (drought stress + 0.75 mM SA + 1 mM proline) treatment, this being 7.9% and 34.4% higher for BRRI dhan66 and BRRI dhan75, respectively, compared to that of rice plants treated with severe drought alone.

### 3.6. Hydrogen Peroxide and Malondialdehyde Content

**Figure 3:**
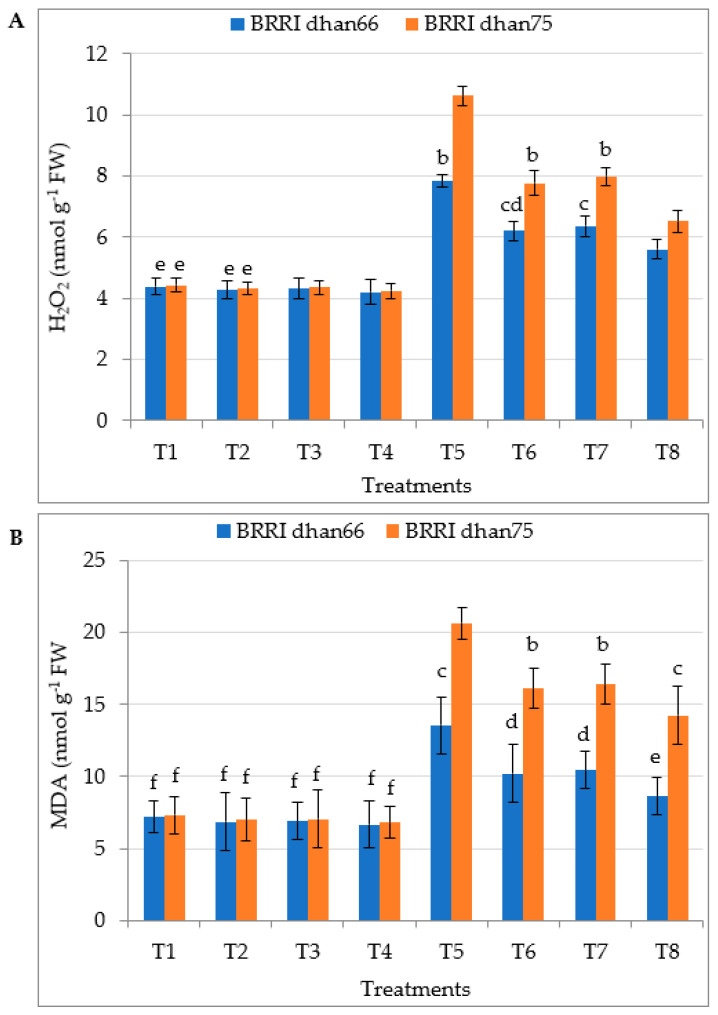
Foliar application of salicylic acid and/or proline regulating (**A**) H_2_O_2_ and (**B**) MDA content of rice leaves under drought stress. The vertical bar indicates the means of five replicates (*n* = 5) and the error bar indicates the standard errors. Different letters indicate significant differences at *p* ≤ 0.05 (Tukey’s test). T1 = control, T2 = 1.5 mM SA, T3 = 2 mM proline, T4 = 0.75 mM SA + 1 mM proline, T5 = 45–50% FC (drought stress), T6 = T5 + 1.5 mM SA, T7 = T5 + 2 mM proline, and T8 = T5 + 0.75 mM SA + 1 mM proline.

Drought stress elevated hydrogen peroxide (H_2_O_2_) accumulation in drought-tolerant BRRI dhan66 and drought-susceptible BRRI dhan75 by 78.6% and 140.3%, respectively, relative to that in the control plants (Figure 3A). On the other hand, the foliar application of SA and/or proline to drought-stressed rice significantly decreased the H_2_O_2_ accumulation of both the rice varieties. However, statistically, the lowest H_2_O_2_ accumulation of drought-stressed BRRI dhan66 (5.61 nmol g^−1^ FW) and BRRI dhan75 (6.53 nmol g^−1^ FW) was observed for the T8 (drought stress + 0.75 mM SA + 1 mM proline) treatment, this being 28.4% and 38.5% lower for BRRI dhan66 and BRRI dhan75, respectively, compared to that of rice plants treated with severe drought alone. 

Drought stress increased the production of malondialdehyde (MDA) of drought-tolerant BRRI dhan66 and drought-susceptible BRRI dhan75 by 89.1% and 182.5%, respectively, compared to that of the control plants (Figure 3B). Due to the amelioration of drought stress through the exogenous application of SA and/or proline, the lowest MDA content of drought-stressed BRRI dhan66 (8.62 nmol g^−1^ FW) and BRRI dhan75 (14.22 nmol g^−1^ FW) was recorded for the T8 (drought stress + 0.75 mM SA + 1 mM proline) treatment, this being 36.3% and 31.1% lower for BRRI dhan66 and BRRI dhan75, respectively, compared to that of rice plants treated with severe drought alone. 

### 3.7. Antioxidant Enzyme Activity

**Figure 4:**
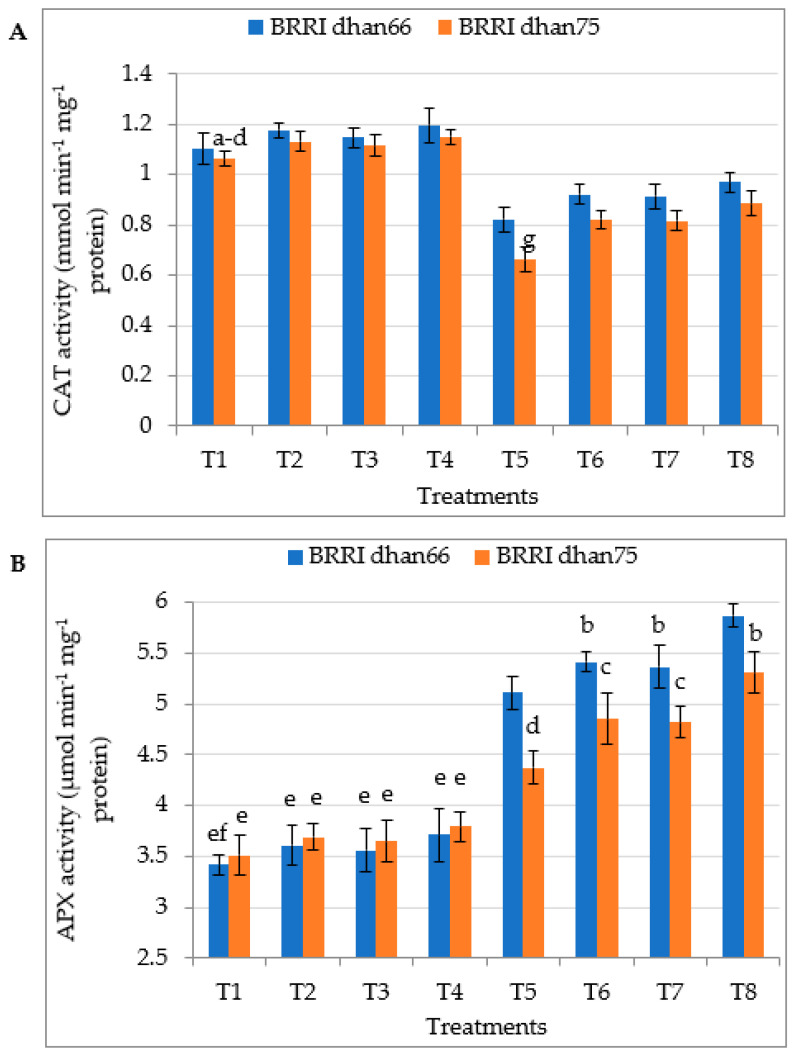

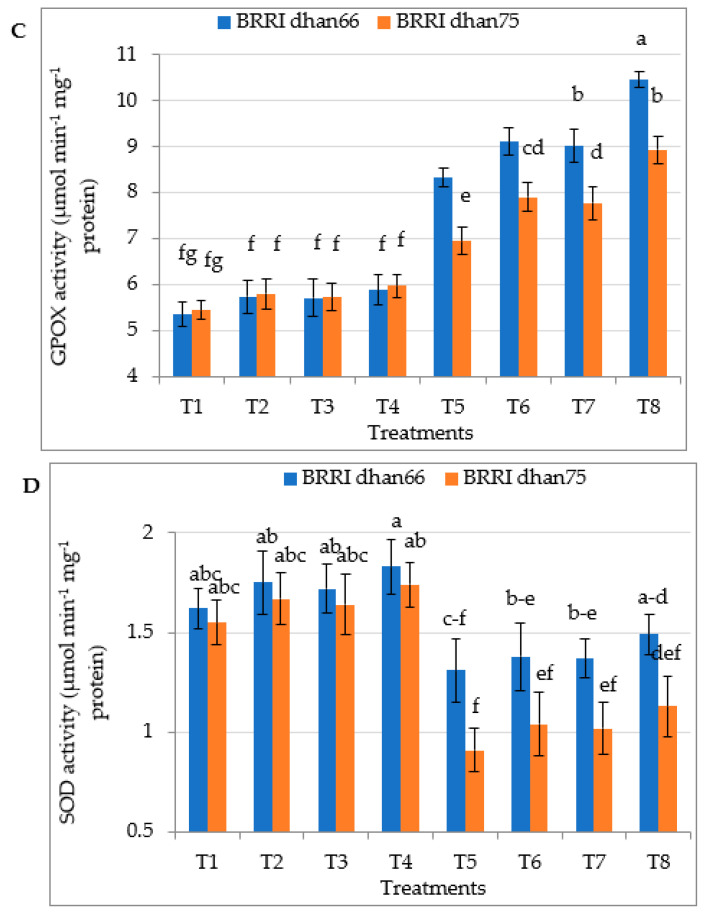
Foliar application of salicylic acid and/or proline enhancing the activity of (**A**) CAT, (**B**) APX, (**C**) GPOX, and (**D**) SOD in two rice varieties under drought stress. The vertical bar indicates the means of five replicates (*n* = 5) and the error bar indicates the standard errors. Different letters indicate significant differences at *p* ≤ 0.05 (Tukey’s test). T1 = control, T2 = 1.5 mM SA, T3 = 2 mM proline, T4 = 0.75 mM SA + 1 mM proline, T5 = 45–50% FC (drought stress), T6 = T5 + 1.5 mM SA, T7 = T5 + 2 mM proline, and T8 = T5 + 0.75 mM SA + 1 mM proline.

Drought stress significantly increased the activity of ascorbate peroxidase (APX) and guaiacol peroxidase (GPX) in both drought-tolerant BRRI dhan66 and drought-susceptible BRRI dhan75 (Figure 4B,C). On the other hand, catalase (CAT) activity and superoxide dismutase (SOD) activity were significantly decreased due to drought stress in both the rice varieties (Figure 4A,D). The pretreatment with SA and/or proline of drought-stressed plants significantly increased CAT, APX, GPX and SOD activity in both the varieties. However, the highest CAT, APX, GPX and SOD activity of drought-stressed BRRI dhan66 at T5 and BRRI dhan75 were recorded for the T8 (drought stress + 0.75 mM SA + 1 mM proline) treatment. 

### 3.8. Nutrients Concentrations

The data in Table 5 reveal that drought stress significantly decreased the nutrient concentrations (root and shoot N, P and K concentrations) of both the rice varieties, compared to those of the control plants. This deleterious effect of drought on nutrient concentrations was ameliorated via the exogenous application of SA and/or proline. However, the highest N, P and K concentrations in the roots and shoots of drought-stressed BRRI dhan66 (1.153% N, 0.1530% P, and 0.2420 K in roots; 0.3860% N, 0.0690% P, and 0.7480% K in shoots) and BRRI dhan75 (0.997% N, 0.1390% P, and 0.2310 K in roots; 0.2830% N, 0.0520% P, and 0.6820% K in shoots) were recorded for the T8 (drought stress + 0.75 mM SA + 1 mM proline) treatment.

## 4. Discussion

### 4.1. Growth and Biomass of Rice 

Drought stress significantly reduced the growth and biomass of both the rice varieties, while the exogenous application of salicylic acid (SA) and/or proline mitigated the stress-imposed adverse effects (Table 1), reduction in crop biomass being one of the main drought stress indicators [16]. Drought stress disrupts developmental processes, leaf areas, photosynthetic rates and metabolic activities [44]. Drought stress inhibits cell elongation, cell division, and nutrient uptake to plants, hence reducing the growth and biomass of crops [45]. Due to the decrease in the water potential gradient between the external and internal environment of crops, water stress appears to reduce the absorption and utilization of water which likely impairs nutrient uptake, consequently impairing crop growth [46], which is reflected by lower tiller formation and poor yield (Table 2). It has been found that crop growth inhibition under drought stress was reduced via the exogenous application of SA or proline [28,29,47]. The exogenous application of SA and/or proline might enhance the water potential gradient and uptake of mineral elements, hence reducing the effects of drought stress on the growth of crops [47]. SA or proline plays a positive role in promoting plant root growth, cell expansion and cell elongation [16,48]. Semida et al. [29] reported that exogenous proline application significantly improved the shoot growth and biomass yield of onion, which might be due to the translocation of proline to roots and the induction of defense responses of onion. It has also reported that exogenous SA application confers drought resistance to wheat by increasing the cell division of roots within the apical meristem [49]. 

### 4.2. Agro-Morphology and Yield of Rice

Drought stress reduced the number of tillers per hill, panicle length, filled grains per panicle and ultimately reduced the seed yield of rice (Table 2), which is supported by Dien et al. [19] and Shukla et al. [50]. Drought stress disrupts leaf gas exchange properties, causes the premature senescence of plant parts, reduces assimilated translocation to growing regions, impairs phloem loading, enhances the abortion of ovules, hence causing spikelet sterility, reduces filled grain and finally lowers yield [51,52,53]. Stomatal closure is the earlier response of water stress to crops, which is regulated by a signaling cascade transported from dehydrated roots to leaves [54,55]. Stomatal closure reduces CO_2_ uptake, which impairs photosynthesis, nutrient uptake, water absorption, and cellular metabolism, finally leading to yield reduction [56]. Drought stress reduced the seed yield per hill of BRRI dhan66 and BRRI dhan75 by 26.9% and 80.2%, respectively, compared to that of the control (Table 2). Our data showed that the exogenous application of SA and proline, individually or combined, significantly enhanced the growth, yield parameters and yield of rice (Table 2) and suggested that SA and/or proline alleviated the harmful effects of drought and increased yield. These results are also in agreement with the result of Semida et al. [29] and Tayyab et al. [47]. It was revealed that both SA and proline has a role in osmotic adjustment that improves root capacity for water uptake [29,57]. The maximization of soil water capture increases stomatal transpiration and leads to higher CO_2_ fixation, which might induce higher biomass production and lead to a higher yield of crops. Both SA and proline increase biomass production and the yield of crops through leaf number and leaf size enlargement, which improve photosynthetic capacity [28]. It has been reported that SA and proline significantly increase the cytokinin content in leaves and grains, improve the absorption and utilization of nitrogen, enhance photosynthetic capacity and increase grain yield [58,59]. 

### 4.3. Gas Exchange Characteristics and Photosynthetic Pigments of Rice

The photosynthetic rate (Pn), stomatal conductance (gs) and transpiration rate (Tr) of both the rice varieties were significantly reduced due to water stress (Table 3). Drought stress reduces stomatal aperture, resulting in reductions in carbon dioxide (CO_2_) influx to leaves, which reduces photosynthesis [15,60,61]. Decreases in turgor pressure enhance stomatal closure, which reduces leaf gas exchange and causes a decrease in CO_2_ assimilation, finally damaging the photosynthetic apparatus [62,63]. Drought stress affects the rubisco activity and PSII structure of the photosystem and inhibits photosynthesis [64]. Drought stress also denatures the proteins involved in photosynthesis [65]. Drought-induced decreases in photosynthetic rates also depend on plant type. The water uptake capacity of the root and photosynthetic capacities are essential factors that reduce the growth and yield of susceptible BRRI dhan75 under drought stress (Table 2; [63]). Chlorophyll and carotenoids are the essential components for photoprotection and the most widely used physiological indicators because they directly affect the efficiency of photosynthesis [66,67]. They are the vital predecessor of photosynthesis, mainly for obtaining light. They act as signaling precursors for the growth of crops under drought stress [68]. Water stress reduces the potential of mesophyll cells to utilize CO_2_, consequently decreasing chlorophyll content [69]. It is reported that a tolerant rice variety maintains a high chlorophyll and carotenoid content and showed better performance under drought stress [70]. Similarly to these findings, our results also showed the higher chlorophyll and carotenoid content and subsequently the highest gs, Tr and Pn in tolerant BRRI dhan66 than those of susceptible BRRI dhan75 (Table 3; Figure 1). The exogenous application of SA and proline, individually or combined, ameliorated the harmful effect of drought and enhanced the chlorophyll and carotenoid content, Pn, gs, and Tr of both the rice varieties. However, s more pronounced impact was found in susceptible BRRI dhan75 for the combined application of SA + proline (Table 3; Figure 1). Both SA and proline help to maintain chlorophyll and carotenoid content and leaf turgor and enhance stomatal conductance which is associated with drought tolerance [71,72]. They increase membrane stability, leaf area, RWC and enhance the rate of photosynthesis, conferring drought tolerance to crops (Table 3, Figure 2; [73]). 

### 4.4. Relative Water Content and Membrane Stability Index 

Water deficit stress reduced the relative water content (RWC) and membrane stability index (MSI) of both tolerant BRRI dhan66 and susceptible BRRI dhan75 (Table 3; Figure 2). Rice is highly sensitive to water stress. Roots absorb less water under water stress conditions than they do under normal conditions. The negative impact of drought on RWC might be due to the reduction in water flow. Reductions in water flow enhance the dehydration of the protoplasm, lead to oxidative damage to chloroplasts, reduce cell membrane integrity (MSI), induce stomatal closure, and decrease CO_2_ concentrations in the mesophyll cells and photo assimilates [29,30]. The elimination of water from the membrane under water stress causes damage to and the dislocation of the lipid structure which decreases MSI [72]. However, the application of SA and proline, individually or combined, increased RWC and MSI in both rice varieties (Table 3; Figure 2). These results suggest that through the activation of the plant defense system, SA and/or proline help the plant to adjust the water relations and membrane functions under water stress conditions. It has been reported that different metabolic responses are generated by SA and proline which affect different plant functions including plant water relations and membrane integrity [28,29,73,74]. It is also reported that SA and proline contribute to osmotic adjustment, and maintain membrane stability and the structure of proteins and enzymes [30]. 

### 4.5. Proline, Soluble Sugar and Starch Content

Plants develop numerous adaptive mechanisms to retain cell turgor pressure under abiotic stress [29,72]. In the present study, the content of proline, soluble sugar and starch showed different variations. Proline content was significantly increased under drought stress. The highest proline accumulation that was found in drought-tolerant BRRI dhan66 might have been related to its higher drought resistance (Table 4). Under water stress conditions, a higher accumulation of proline is one of the most effective mechanisms of osmotic regulation and of alleviating drought stress [29,47]. Plant water status might be imbalanced under drought stress which disrupts osmotic adjustment and finally leads to a higher accumulation of compatible osmolytes in crops. Proline has antioxidant activity which thereby reduces lipid peroxidation and promotes cell homeostasis by protecting the redox balance [75]. Drought stress decreased the soluble sugar content of BRRI dhan66 and BRRI dhan75 by 19.0% and 32.8%, respectively, and starch content by 9.7% and 35.5%, respectively, compared to those of control plants (Table 4). Several studies have shown that soluble sugar and starch contents increase under drought stress. It is reported that plants mitigate drought-induced damage via the higher accumulation of soluble sugar and starch. However, continuous water shortages due to prolonged drought will destroy the structure of plants and affect the synthesis of proteins and sugars [75,76]. Siaut et al. [77] explained that starch is degraded to provide energy and carbon when photosynthesis is limited under prolonged water stress conditions. Exogenous applications of SA and/or proline significantly increased the RWC and proline, soluble sugar and starch content under water stress, indicating their role in the restoration of water in plant tissue and thus osmoprotection. Salicylic acid (SA) and/or proline application might contribute to the up-regulation of proline, soluble sugar and starch biosynthesis, hence increasing their contents. It was found that SA and/or proline application enhances the water capture capacity of roots, leads to the presence of more nutrients and enhances photosynthesis, which might be enhanced via proline, soluble sugar and starch biosynthesis (Table 3, Table 4 and Table 5 [29,47]). The exogenous application of SA and/or proline up-regulate pyrroline-5-carboxylate synthase which is involved in proline synthesis and down-regulates the proline dehydrogenase gene which enhances cell death [73,78]. In the present research, drought-tolerant BRRI dhan66 showed a higher proline, soluble sugar and starch accumulation ability than that of drought-susceptible BRRI dhan75 under the same water stress conditions (Table 4). These results suggest that proline, soluble sugar and starch accumulation in rice under drought stress depended not only on the severity of drought stress but also on the characteristics of the different varieties. In addition, a higher osmolyte accumulation ability was reported to contribute to higher drought tolerance in rice [19,72]. Therefore, a higher osmolyte accumulation ability might be used as an indicator for drought tolerance potential in rice and this ability is increased through exogenous SA and/or proline application. 

### 4.6. Hydrogen Peroxide and Malondialdehyde Content

The overproduction of reactive oxygen species (ROS) and malondialdehyde (MDA) under drought stress are the indicators of oxidative stress [47,72,79]. An over-reduction in the electron transport chain of mitochondria and chloroplasts under water stress leads to the overproduction of ROS [80]. MDA is produced via lipid peroxidation under drought stress, while ROS causes the lipid peroxidation of plant membranes under drought [47,81]. It is reported that hydrogen peroxide (H_2_O_2_) is a highly toxic radical that damages different cell components and proteins, and enhances lipid peroxidation and membrane damage, finally leading to cell death [82]. It is also reported that the overproduction of H_2_O_2_ and MDA causes them to react with proteins, lipids and deoxyribonucleic acid and causes oxidative damage to crops [83]. A higher production of H_2_O_2_ and MDA increased electrolyte leakage through inducing decreases in the cell membrane integrity of plants under drought stress (Figure 2 and Figure 3 [30,50]). They cause membrane disorganization and metabolic toxicity, resulting in a higher leakage of solutes. Furthermore, drought-treated higher electrolyte leakage might be the reason for cell damage and consequent osmotic suffering. It has been shown that osmolytes maintain turgor pressure and enhance water uptake capacity by increasing osmotic pressure in the cytoplasm [84]. Osmolytes, other than being involved in osmotic adjustment, also play a role as scavengers of ROS and MDA by increasing the activities of enzymes, hence helping to mitigate the adverse effects of oxidative stress [85,86]. These reports support our results, which showed that water stress significantly increased the accumulation of H_2_O_2_ and MDA, while the exogenous application of SA and/or proline significantly decreased their accumulation in the drought-stressed rice plants (Figure 3). Similar findings were also observed by other researchers who found that the application of SA and proline significantly reduced the level of H_2_O_2_ and MDA in different plants under stress [47,87]. Furthermore, significant differences in H_2_O_2_ and MDA accumulation as well as osmolyte accumulation were observed between the drought-tolerant and drought-susceptible variety. The lowest values of H_2_O_2_ and MDA found in the tolerant variety might have been due to the higher accumulation of osmolytes. 

### 4.7. Antioxidant Enzyme Activities

Plants possess enzymatic and non-enzymatic antioxidant defense systems, which regulate oxidative stress via the scavenging of ROS and protect plant cells form oxidative damage [47,72]. CAT is a heme-containing enzyme and minimizes the overproduction of H_2_O_2_ under oxidative stress through converting H_2_O_2_ into H_2_O and O_2_ [88]. The ascorbate–glutathione pathway is a major H_2_O_2_-detoxifying system in plant cells, in which H_2_O_2_ is reduced by APX where ascorbate acts as an electron donor [89]. For rice, under drought stress, the activities of these enzymes are considered one of the most important protective mechanisms against oxidative stress [83]. GPX not only scavenges ROS but also produces related compounds such as lignin, guaiacol and payragallol. These compounds work as electron donors for scavenging H_2_O_2_ both intra- and extra-cellularly [90]. SOD acts as the primary defense line against drought-induced oxidative stress [80]. In the present study, APX and GPX showed increasing trends, while CAT and SOD showed decreasing trends under water stress conditions (Figure 4), which is similar to the findings of other studies [16,75,80,90]. CAT and SOD activities were significantly lower in rice varieties, which might have been due to prolonged drought stress. Gao et al. [75] reported that the activities of CAT and SOD increase under short-term water stress, while they decrease under prolonged water stress. It is also reported that the effectiveness of the activity of antioxidant enzyme depends on plant species, and the severity and duration of drought stress [28]. Increase in APX and GPX are not enough to neutralize the overproduction of H_2_O_2_ under prolonged drought stress. It has been observed that the exogenous application of SA or proline up-regulates and increases the activities of CAT, APX, GPX and SOD in different plant species under water stress [27,28,30,91]. Similar to a previous study, in the present study, the exogenous application of SA and/or proline augmented the activity of CAT, APX, GPX and SOD, consequently decreasing the level of H_2_O_2_ as well as MDA in water-stressed plants (Figure 3 and Figure 4). These findings cannot be explained in view of the argument of Ghafoor et al. [92] that the exogenous application of proline cannot control enhanced levels of stress by elevating antioxidant enzyme activities. Effects of proline on the activity of antioxidant enzymes may be plant species- as well as dose-dependent [32]. In this study, drought-tolerant and drought-susceptible varieties differed considerably in terms of the activity of different antioxidant enzymes under drought stress and also under exogenous SA or proline application (Figure 4). 

### 4.8. Nutrients Concentrations 

Mineral elements are crucial for the growth, development, and survival of crops under stress. N, P and K^+^ are important inorganic nutrients and they play multiple essential roles in plant metabolism. They play an important role in osmoregulation, cellular energy transfer, respiration and photosynthesis [23,28,93]. However, the optimum concentration range of each of these nutrients should be maintained for the proper growth and development of plants. On the other hand, Table 5 shows that drought stress significantly reduced the N, P and K^+^ concentrations in the roots and shoots of both the rice varieties, while the exogenous application of SA and/or proline decreased drought stress effects, hence enhancing nutrient concentrations in the roots and shoots of both drought-stressed rice varieties. However, the highest N, P ad K^+^ concentrations in roots and shoots was observed due to the combined application of SA + proline. It has been reported that exogenous SA or proline application enhances the uptake of N, P and K^+^ under drought stress, hence maintaining the nutrient status, growth and development of plants [23,28]. It has been reported that K^+^ maintains cell turgor and stomatal movement, and maintains water balance in plants [23,94]. Prolonged drought stress disrupts the plant root system and decreases potassium uptake from the soil. Drought stress also enhances lipid peroxidation which enhances the leakage of K^+^ from cells [23]. Many studies have shown that exogenous SA or proline application protects membranes by reducing lipid peroxidation under osmotic stress and reducing K^+^ leakage from cells [23,94]. 

## 5. Conclusions

This study exposed that prolonged drought stress significantly decreased the plant growth, yield, gas exchange characteristics, photosynthetic pigments, relative water content (RWC), membrane stability index, soluble sugar content, starch content, and uptake of nutrients in roots and shoots. Drought stress also increased the accumulation of reactive oxygen species which enhanced lipid peroxidation (MDA). Th exogenous application of SA (1.5 mM) or proline (2 mM) separately reduced the harmful effects of drought. However, their combined application (0.75 mM SA + 1 mM proline) was found to be more effective than was their separate application. The combined application of SA + proline more effectively alleviated the harsh effects of drought-induced oxidative stress through a higher accumulation of osmolytes and the up-regulation of antioxidant enzyme activities. Consequently, their combined application enhanced gas exchange characteristics, photosynthetic pigments, RWC, the nutrient uptake capacity and yield of rice more pronouncedly than did their single application. Our results suggest that the application of SA and/or proline prevents the harmful impacts of drought-induced oxidative and osmotic stress on rice by modulating its physiological attributes, biochemical parameters, and antioxidant enzyme activity. 

## Figures and Tables

**Table 1:** Exogenous application of salicylic acid and/or proline enhanced growth and biomass of rice under drought stress.

Variety	Treatment	Plant Height (cm)	Fresh Weight (g)/Plant	Dry Weight (g)/Plant
BRRI dhan66	T1	58.8 ± 2.15 bc	0.836 ± 0.03 a	0.152 ± 0.02 abc
	T2	60.4 ± 1.99 ab	0.851 ± 0.01 a	0.156 ± 0.01 ab
	T3	60.1 ± 1.99 ab	0.843 ± 0.03 a	0.155 ± 0.01 ab
	T4	62.4 ± 2.74 a	0.862 ± 0.04 a	0.159 ± 0.00 a
	T5	53.9 ± 1.78 de	0.646 ± 0.01 c	0.133 ± 0.01 efg
	T6	55.2 ± 2.83 de	0.712 ± 0.03 b	0.139 ± 0.02 cdef
	T7	54.7 ± 1.77 de	0.701 ± 0.07 bc	0.138 ± 0.02 def
	T8	56.7 ± 2.70 cd	0.743 ± 0.03 b	0.143 ± 0.01 bcde
BRRI dhan75	T1	53.7 ± 1.37 e	0.817 ± 0.01 a	0.151 ± 0.03 abc
	T2	55.5 ± 1.41 de	0.831 ± 0.06 a	0.155 ± 0.01 ab
	T3	55.7 ± 1.44 de	0.824 ± 0.04 a	0.155 ± 0.01 ab
	T4	56.7 ± 2.34 cd	0.84 ± 0.03 a	0.158 ± 0.02 a
	T5	37.9 ± 2.08 h	0.271 ± 0.02 f	0.103 ± 0.01 i
	T6	43.8 ± 1.23 g	0.473 ± 0.02 e	0.121 ± 0.02 gh
	T7	43.6 ± 1.44 g	0.436 ± 0.05 e	0.119 ± 0.01 g
	T8	47.6 ± 2.53 f	0.551 ± 0.02 d	0.128 ± 0.01 fgh

Mean values followed by different letters in the same column are significantly different from each other (Tukey’s test, *p* < 0.05). The data represent the means of five replicates ± the standard error (*n* = 5). T1 = control, T2 = 1.5 mM SA, T3 = 2 mM proline, T4 = 0.75 mM SA + 1 mM proline, T5 = 45–50% FC (drought stress), T6 = T5 + 1.5 mM SA, T7 = T5 + 2 mM proline, and T8 = T5 + 0.75 mM SA + 1 mM proline.

**Table 2:** Exogenous application of salicylic acid and/or proline enhanced different yield-contributing characteristics and yield of rice under drought stress.

Variety	Treatment	Plant Height (cm)	Total Tillers per Hill	Effective Tillers per Hill	Panicle Length (cm)	Filled Grain per Panicle	Unfilled Grain per Panicle	Thousand-Seed Weight (g)	Seed Yield (g) per Hill
BRRI dhan66	T1	116.3 ± 3.51 ab	15.7 ± 0.58 ab	14.3 ± 0.36 abc	25.1 ± 0.65 abc	177.7 ± 3.51 bc	32.3 ± 1.15 ghi	22.1 ± 1.06 ab	21.5 ± 0.51 a
	T2	118.5 ± 4.21 a	16.3 ± 0.41 a	14.7 ± 0.58 ab	26.1 ± 1.01 ab	185.3 ± 4.13 a	29.3 ± 1.76 hi	22.5 ± 0.50 a	21.8 ± 1.03 a
	T3	117.8 ± 3.30 a	15.7 ± 0.58 ab	14.7 ± 0.65 ab	25.9 ± 1.02 ab	182.3 ± 4.41 abc	31.7 ± 1.10 ght	22.3 ± 0.55 ab	21.7 ± 1.25 a
	T4	119.3 ± 3.51 a	16.7 ± 1.53 a	15.0 ± 1.50 a	26.3 ± 2.33 a	187.2 ± 4.00 a	28.1 ± 1.87 i	22.6 ± 1.30 a	22.1 ± 1.13 a
	T5	107.0 ± 2.65 d	13.7 ± 1.00 bc	12.0 ± 1.00 c	21.3 ± 0.95 d	146.3 ± 2.89 f	78.0 ± 1.73 d	19.8 ± 0.73 b	15.7 ± 0.93 d
	T6	110.2 ± 3.02 cd	14.7 ± 0.58 ab	13.0 ± 0.58 abc	21.9 ± 0.62 d	160.7 ± 4.16 de	64.3 ± 1.53 e	20.6 ± 0.54 ab	17.5 ± 1.45 cd
	T7	109.3 ± 4.93 cd	14.7 ± 0.63 ab	12.3 ± 1.13 bc	21.7 ± 0.66 d	157.3 ± 3.30 e	68.3 ± 1.53 e	20.6 ± 0.28 ab	17.1 ± 1.20 cd
	T8	112.7 ± 3.06 bc	15.3 ± 1.26 ab	13.3 ± 1.00 abc	22.3 ± 1.53 d	166.3 ± 3.62 d	57.4 ± 2.46 f	21.3 ± 1.70 ab	18.7 ± 0.81 bc
BRRI dhan75	T1	108.3 ± 3.11 cd	14.7 ± 0.58 ab	13.3 ± 0.58 abc	22.9 ± 0.85 cd	176.0 ± 2.65 c	35.3 ± 1.15 g	20.6 ± 0.89 ab	21.0 ± 0.50 ab
	T2	110.3 ± 3.79 cd	15.0 ± 1.00 ab	13.7 ± 1.09 abc	23.6 ± 0.52 bcd	184.3 ± 3.21 ab	34.3 ± 1.53 gh	20.9 ± 0.49 ab	21.4 ± 1.36 a
	T3	109.5 ± 4.82 cd	15.0 ± 1.15 ab	13.7 ± 1.53 abc	23.5 ± 0.50 bcd	182.4 ± 4.87 abc	34.0 ± 2.00 gh	20.7 ± 0.57 ab	21.2 ± 1.24 a
	T4	111.4 ± 4.50 cd	15.3 ± 1.57 ab	14.1 ± 1.46 abc	23.9 ± 1.84 abcd	187.3 ± 4.00 a	33.6 ± 2.04 gh	21.3 ± 0.87 ab	21.7 ± 1.52 a
	T5	63.7 ± 2.52 f	8.3 ± 1.00 e	5.7 ± 0.61 e	11.6 ± 1.30 f	56.3 ± 2.89 i	146.3 ± 4.21 a	13.0 ± 0.70 d	4.2 ± 0.32 g
	T6	79.3 ± 2.63 e	10.3 ± 1.16 de	7.3 ± 0.40 de	14.7 ± 0.53 e	78.3 ± 3.06 h	105.3 ± 3.13 c	15.9 ± 0.43 c	7.7 ± 0.35 f
	T7	77.3 ± 2.52 e	10.3 ± 0.72 de	7.0 ± 0.00 de	14.4 ± 0.58 e	71.7 ± 3.51 h	111.6 ± 3.21 b	15.5 ± 0.35 cd	7.3 ± 0.32 f
	T8	81.6 ± 4.13 e	11.6 ± 1.06 cd	8.7 ± 0.76 d	16.4 ± 1.30 e	114.7 ± 5.03 g	81.6 ± 3.90 d	16.7 ± 0.80 c	9.6 ± 0.210 e

Mean values followed by different letters in the same column are significantly different from each other (Tukey’s test, *p* < 0.05). The data represent the means of five replicates ± the standard error (*n* = 5). T1 = control, T2 = 1.5 mM SA, T3 = 2 mM proline, T4 = 0.75 mM SA + 1 mM proline, T5 = 45–50% FC (drought stress), T6 = T5 + 1.5 mM SA, T7 = T5 + 2 mM proline, and T8 = T5 + 0.75 mM SA + 1 mM proline.

**Table 3:** Exogenous application of salicylic acid and/or proline enhanced gas exchange attributes and relative water content of rice under drought stress.

Variety	Treatment	Photosynthetic Rate (µmol m^−2^ s^−1^)	Stomatal Conductance (mmol m^−2^ s^−1^)	Transpiration Rate (mmol m^−2^ s^−1^)	Relative Water Content
BRRI dhan66	T1	15.7 ± 0.50 a	0.414 ± 0.02 a	6.58 ± 0.37 a	90.6 ± 2.51 a
	T2	16.2 ± 0.83 a	0.426 ± 0.03 a	6.81 ± 0.46 a	93.8 ± 3.02 a
	T3	16.1 ± 1.08 a	0.421 ± 0.02 a	6.74 ± 0.50 a	93.3 ± 2.53 a
	T4	16.4 ± 1.35 a	0.437 ± 0.05 a	6.95 ± 0.46 a	94.4 ± 3.64 a
	T5	11.2 ± 0.84 e	0.276 ± 0.03 cd	4.06 ± 0.20 bcd	70.4 ± 1.95 f
	T6	13.4 ± 0.40 cd	0.313 ± 0.02 bc	4.62 ± 0.40 bc	81.4 ± 3.00 cd
	T7	13.1 ± 0.27 d	0.305 ± 0.03 bc	4.48 ± 0.52 bc	80.1 ± 3.57 cd
	T8	13.8 ± 0.80 bcd	0.351 ± 0.03 b	5.04 ± 0.52 b	84.3 ± 3.25 bc
BRRI dhan75	T1	15.1 ± 0.35 abc	0.411 ± 0.01 a	6.46 ± 0.30 a	88.9 ± 3.72 ab
	T2	15.5 ± 1.08 ab	0.422 ± 0.02 a	6.68 ± 0.31 a	92.7 ± 1.95 a
	T3	15.5 ± 0.60 ab	0.419 ± 0.02 a	6.57 ± 0.41 a	91.7 ± 2.05 a
	T4	15.7 ± 1.09 a	0.430 ± 0.03 a	6.81 ± 0.36 a	92.4 ± 3.44 a
	T5	6.1 ± 0.42 g	0.106 ± 0.00 f	1.89 ± 0.06 f	57.3 ± 1.05 g
	T6	9.3 ± 0.62 f	0.186 ± 0.01 e	3.13 ± 0.20 de	71.2 ± 2.17 ef
	T7	9.2 ± 0.78 f	0.175 ± 0.03 e	2.91 ± 0.19 ef	70.4 ± 2.39 f
	T8	10.5 ± 0.62 ef	0.229 ± 0.03 de	3.65 ± 0.46 cde	76.6 ± 3.20 de

Mean values followed by different letters in the same column are significantly different from each other (Tukey’s test, *p* < 0.05). The data represent the means of five replicates ± the standard error (*n* = 5). T1 = control, T2 = 1.5 mM SA, T3 = 2 mM proline, T4 = 0.75 mM SA + 1 mM proline, T5 = 45–50% FC (drought stress), T6 = T5 + 1.5 mM SA, T7 = T5 + 2 mM proline, and T8 = T5 + 0.75 mM SA + 1 mM proline.

**Table 4:** Foliar application of salicylic acid and/or proline regulates proline, soluble sugar and starch content of rice under drought stress.

Variety	Treatment	Proline Content (µg g^−1^ FW)	Soluble Sugar Content (mg g^−1^ FW)	Starch Content (mg g^−1^ FW)
BRRI dhan66	T1	1.36 ± 0.06 g	31.6 ± 1.30 ab	15.9 ± 0.95 abcd
	T2	1.41 ± 0.19 g	32.7 ± 2.37 a	16.4 ± 1.40 ab
	T3	1.39 ± 0.22 g	32.5 ± 2.46 a	16.2 ± 1.20 ab
	T4	1.45 ± 0.15 g	32.8 ± 2.18 a	16.7 ± 1.18 a
	T5	3.75 ± 0.21 cd	25.6 ± 1.70 d	14.4 ± 1.36 ef
	T6	4.12 ± 0.24 ab	27.1 ± 2.20 d	15.1 ± 1.14 cde
	T7	4.06 ± 0.17 abc	26.9 ± 1.10 d	14.9 ± 1.21 de
	T8	4.25 ± 0.39 a	29.6 ± 2.40 c	15.5 ± 1.35 bcd
BRRI dhan75	T1	1.32 ± 0.25 g	30.2 ± 1.60 bc	16.0 ± 1.32 abc
	T2	1.37 ± 0.20 g	31.1 ± 1.95 abc	16.5 ± 1.00 a
	T3	1.34 ± 0.29 g	31.1 ± 1.15 abc	16.4 ± 1.25 ab
	T4	1.41 ± 0.20 g	31.5 ± 1.41 ab	16.7 ± 1.18 a
	T5	2.88 ± 0.25 f	20.3 ± 1.15 f	10.3 ± 0.88 h
	T6	3.41 ± 0.34 de	23.1 ± 1.36 e	12.1 ± 0.67 g
	T7	3.37 ± 0.20 e	22.8 ± 1.11 e	11.9 ± 0.79 g
	T8	3.78 ± 0.21 bc	25.3 ± 1.25 d	13.9 ± 0.89 f

Mean values followed by different letters in the same column are significantly different from each other (Tukey’s test, *p* < 0.05). The data represent the means of five replicates ± the standard error (*n* = 5). T1 = control, T2 = 1.5 mM SA, T3 = 2 mM proline, T4 = 0.75 mM SA + 1 mM proline, T5 = 45–50% FC (drought stress), T6 = T5 + 1.5 mM SA, T7 = T5 + 2 mM proline, and T8 = T5 + 0.75 mM SA + 1 mM proline.

**Table 5:** Foliar application of salicylic acid and/or proline regulates root and shoot nutrient concentrations of rice under drought stress.

Variety	Treatment	%N		%P		%K	
		Roots	Shoots	Roots	Shoots	Roots	Shoots
BRRI dhan66	T1	1.183 ± 0.03 a	0.4072 ± 0.01 a	0.1557 ± 0.01 cd	0.0778 ± 0.00 c	0.2447 ± 0.02 bc	0.8217 ± 0.01 de
	T2	1.185 ± 0.06 a	0.4120 ± 0.03 a	0.1590 ± 0.01 ab	0.0789 ± 0.00 bc	0.2480 ± 0.01 ab	0.8320 ± 0.02 ab
	T3	1.184 ± 0.04 a	0.410 ± 0.03 a	0.1583 ± 0.01 abc	0.0788 ± 0.00 bc	0.2470 ± 0.00 abc	0.8310 ± 0.03 ab
	T4	1.192 ± 0.03 a	0.4170 ± 0.04 a	0.1599 ± 0.03 a	0.0791 ± 0.00 bc	0.2510 ± 0.00 a	0.8350 ± 0.01 a
	T5	1.112 ± 0.01 d	0.3650 ± 0.1 c	0.1465 ± 0.02 e	0.0613 ± 0.00 f	0.2360 ± 0.01 ef	0.7260 ± 0.01 h
	T6	1.135 ± 0.01 c	0.3750 ± 0.01 bc	0.1490 ± 0.02 e	0.0650 ± 0.00 e	0.2390 ± 0.02 de	0.7420 ± 0.01 fg
	T7	1.132 ± 0.03 c	0.3720 ± 0.01 bc	0.1480 ± 0.01 e	0.0640 ± 0.00 ef	0.2370 ± 0.03 de	0.7390 ± 0.02 g
	T8	1.153 ± 0.01 b	0.3860 ± 0.01 b	0.1530 ± 0.01 d	0.0690 ± 0.00 d	0.2420 ± 0.01 cd	0.7480 ± 0.03 f
BRRI dhan75	T1	1.181 ± 0.05 a	0.4058 ± 0.03 a	0.1570 ± 0.02 bc	0.0811 ± 0.01 ab	0.2450 ± 0.02 bc	0.8157 ± 0.03 e
	T2	1.184 ± 0.03 a	0.4110 ± 0.04 a	0.1598 ± 0.03 a	0.0825 ± 0.00 a	0.2481 ± 0.02 ab	0.8260 ± 0.02 bcd
	T3	1.183 ± 0.06 a	0.4090 ± 0.04 a	0.1593 ± 0.03 ab	0.0823 ± 0.01 a	0.2476 ± 0.00 ab	0.8240 ± 0.02 cd
	T4	1.189 ± 0.02 a	0.4160 ± 0.03 a	0.1606 ± 0.01 a	0.0826 ± 0.00 a	0.2492 ± 0.01 ab	0.8290 ± 0.01 abc
	T5	0.938 ± 0.01 g	0.2058 ± 0.02 f	0.1240 ± 0.01 i	0.0336 ± 0.00 i	0.2161 ± 0.02 i	0.5705 ± 0.01 k
	T6	0.971 ± 0.04 f	0.2510 ± 0.01 e	0.1310 ± 0.01 g	0.0410 ± 0.00 h	0.2260 ± 0.00 gh	0.6130 ± 0.01 j
	T7	0.965 ± 0.01 f	0.2430 ± 0.02 e	0.1280 ± 0.01 h	0.0380 ± 0.00 h	0.2230 ± 0.01 h	0.6110 ± 0.02 j
	T8	0.997 ± 0.02 e	0.2830 ± 0.02 d	0.1390 ± 0.02 f	0.0520 ± 0.00 g	0.2310 ± 0.01 fg	0.6820 ± 0.03 i

Mean values followed by different letters in the same column are significantly different from each other (Tukey’s test, *p* < 0.05). The data represent the means of five replicates ± the standard error (*n* = 5). T1 = control, T2 = 1.5 mM SA, T3 = 2 mM proline, T4 = 0.75 mM SA + 1 mM proline, T5 = 45–50% FC (drought stress), T6 = T5 + 1.5 mM SA, T7 = T5 + 2 mM proline, and T8 = T5 + 0.75 mM SA + 1 mM proline.

## Data Availability

The data are contained within the article.

## Supplementary Materials

The following supporting information can be downloaded at https://www.mdpi.com/article/10.3390/antiox12071438/s1. Table S1: Exogenous application of different concentrations of salicylic acid and/or proline on growth and biomass of rice under drought stress.
