# Supplementary material for: Combined Effect of Salicylic Acid and Proline Mitigates Drought Stress in Rice (Oryza sativa L.) through the Modulation of Physiological Attributes and Antioxidant Enzymes
Source: Antioxidants (Basel). 2023 Jul 18;12(7):1438. doi: 10.3390/antiox12071438 (PMC10375981; doi:10.3390/antiox12071438)
Supplement: Supplementary file 1 [file antioxidants-12-01438-s001.zip › antioxidants-2467578-supplementary.pdf]

antioxidants

Supplementary Materials: The following supporting information can be downloaded at: www.mdpi.com/article/10.3390/antiox12071438/s1.

Table S1. Exogenous application of different concentration of salicylic acid and/or proline on growth and biomass of rice under drought stress.

Variety	Treatment		Plant height (cm)	Fresh Weight (g)/Plant	Total Chlorophyll (mg g^{-1} FW)
BRRI dhan66	Control	T1	38.6 ± 1.69 a	0.471 ± 0.02 a	4.72 ± 0.31 a
	Drought (D)	T2	34.9 ± 2.55 d	0.381 ± 0.03 f	4.01 ± 0.42 d
	D + 1 mM SA	T3	35.8 ± 2.16 c	0.414 ± 0.02 d	4.22 ± 0.51 c
	D + 1.5 mM SA	T4	36.7 ± 2.15 b	0.441 ± 0.01 bc	4.51 ± 0.29 ab
	D + 2 mM SA	T5	36.1 ± 1.58 b	0.426 ± 0.03 cd	4.31 ± 0.36 bc
	D + 1 mM Pro	T6	35.5 ± 1.95 c	0.408 ± 0.03 de	4.18 ± 0.41 c
	D + 2 mM Pro	T7	36.4 ± 2.31 b	0.431 ± 0.03 c	4.45 ± 0.51 b
	D + 3 mM Pro	T8	35.8 ± 2.44 c	0.418 ± 0.02 d	4.28 ± 0.61 bc
	D + (1.5 mM SA + 2 mM Pro)	T9	34.3 ± 2.71 d	0.408 ± 0.02 de	3.89 ± 0.22 e
	D + (1 mM SA + 1 mM Pro)	T10	36.8 ± 1.91 b	0.446 ± 0.03 b	4.48 ± 0.31 b
	D + (0.75 mM SA + 1 mM Pro)	T11	37.3 ± 1.28 ab	0.449 ± 0.02 b	4.69 ± 0.33 a
BRRI dhan75	Control	T1	35.2 ± 2.41 c	0.452 ± 0.01 b	4.88 ± 0.51 a
	Drought (D)	T2	26.7 ± 1.62 i	0.294 ± 0.01 i	3.16 ± 0.28 h
	D + 1 mM SA	T3	28.6 ± 1.74 gh	0.344 ± 0.03 h	3.58 ± 0.45 fg
	D + 1.5 mM SA	T4	31.5 ± 2.13 f	0.381 ± 0.02 f	3.92 ± 0.41 de
	D + 2 mM SA	T5	29.4 ± 1.88 g	0.358 ± 0.02 gh	3.75 ± 0.38 f
	D + 1 mM Pro	T6	28.2 ± 2.42 gh	0.338 ± 0.03 h	3.51 ± 0.31 fg
	D + 2 mM Pro	T7	30.6 ± 2.18 fg	0.369 ± 0.02 g	3.84 ± 0.45 e
	D + 3 mM Pro	T8	29.0 ± 1.85 g	0.351 ± 0.02 gh	3.68 ± 0.52 f
	D + (1.5 mM SA + 2 mM Pro)	T9	27.3 ± 2.66 g	0.331 ± 0.03 h	3.29 ± 0.42 g
	D + (1 mM SA + 1 mM Pro)	T10	30.9 ± 2.43 fg	0.373 ± 0.04 fg	3.98 ± 0.35 d
	D + (0.75 mM SA + 1 mM Pro)	T11	33.8 ± 2.71 e	0.394 ± 0.03 e	4.15 ± 0.38

Mean values followed by different letters in the same column are significantly different from each other (Tukey's test $P < 0.05$). The data represent means of five replicates ± standard error ($n = 5$).
